# Relatively higher norms of blood flow velocity of major intracranial arteries in North-West Iran

**DOI:** 10.1186/1756-0500-3-174

**Published:** 2010-06-22

**Authors:** M Farhoudi, SN Kermani, H Sadeghi-Bazargani

**Affiliations:** 1 Neuroscience Research Center (NSRC), Tabriz University of Medical Sciences, Tabriz, Iran; 2 Faculty of Medicine, Tabriz University of Medical Sciences, Tabriz, Iran

## Abstract

**Background:**

Transcranial Doppler (TCD) is a noninvasive, less expensive and harmless hemodynamic study of main intracranial arteries. The aim of this study was to assess normal population values of cerebral blood flow velocity and its variation over age and gender in a given population.

**Findings:**

Eighty healthy volunteers including 40 people with an age range of 25-40 years (group1) and 40 persons with an age range of 41-55 years (group2) were studied. In each group 20 males and 20 females were enrolled. Peak systolic, end diastolic and mean velocities of nine main intracranial arteries were determined using TCD. Mean age of the studied volunteers was 31.6 ± 4.50 years in group one and 47.2 ± 4.3 years in group two. Mean age among males was 40 years and among females it was 39. Mean blood flow velocity in middle, anterior and posterior cerebral arteries, vertebral and basilar arteries was 60 ± 8, 52 ± 9, 42 ± 6, 39 ± 8 and 48 ± 8 cm/sec respectively. Cerebral blood flow velocities among females were relatively higher than males. Cerebral blood flow velocity of left side was relatively higher than right side.

**Conclusion:**

Compared to previous studies, cerebral blood flow velocity in this population was relatively higher.

## Background

Transcranial Doppler (TCD) is a noninvasive, less expensive, safe, and harmless technique being commonly used studying main intracranial arteries especially in cerebrovascular occlusive disease. Many factors can affect the cerebral blood flow velocities (CBFV) including age, sex, body temperature, blood viscosity, arterial blood pressure, obesity, metabolic state, cardiac function, carbon dioxide tension, oxygen tension, intracranial pressure, some drugs, smoking and alcohol. Increased flow velocity may be associated with a younger age, anemia, severely increased blood pressure, hypermetabolic states such as hyperthyroidism and anemia, increased carbon dioxide tension or some drugs such as an acetazolamid and mannitol. Decreased CBFV may be found with older age, increased hematocrit and fibrinogen, decreased carbon dioxide tension, increased oxygen tension, increased intracranial pressure or high dose of some drugs such as barbiturates and some physiological changes such as sleeping or awakening and exercise[1-3]. Alteration in hemoglobin level significantly influences TCD measured flow velocities presumably by blood viscosity being inversely related to flow velocity [2,4]. Due to some geographic variations, it is good for each medical center to detect normal blood flow velocity values for the regional population which in turn can help in better interpretation of TCD results. The aim of this study was to assess normal population values of cerebral blood flow velocity and its variation over age and gender in an Iranian population.

## Methods

In a descriptive-analytic study, 80 healthy volunteers were examined with TCD. Fourty men and 40 women in an age range of 25-55 years were enrolled. Each female subject was matched with a male of the same age. The population was divided into two groups: group one was 25 - 40 years (20 males - 20 females) and group two was 41 - 55 years age group (20 males and 20 females). These volunteers lacked any history of cardiovascular risk factors or any other significant diseases. They didn't use any drugs too. Among all volunteers fasting blood sugar, blood urea, hemoglobin and lipid profile (LDL, HDL, Total cholesterol, Triglyceride) were normal. Based on physical examination, hypertensive (systolic blood pressure > 140 mmHg, diastolic blood pressure > 90 mmHg) or obese subjects (body mass index > 30 kg/m ^2^) were excluded. The normal healthy persons were studied on supine position using 2 MHz probe and standard method by transcranial Doppler (Multi- Dop X4, DWL, Germany). Asymptomatic and hemodynamic extracranial internal carotid stenosis were excluded by continue wave Doppler study and suprathrochlear artery response to facial and superficial temporal arteries compression. Then the middle cerebral artery (MCA), anterior cerebral artery (ACA) and posterior cerebral artery segments 1, 2 (PCA_1_, PCA_2_) were studied via temporal acoustic window and the distal vertebral artery segments 3, 4(VA_3_, VA_4_) and basilar artery (BAS) were detected through suboccipital window. The average of the maximum velocity (MV), peak systolic velocity (PV), end diastolic velocity (EDV) and pulsatility index (PI) were automatically calculated and recorded.

## Results

Demographic and laboratory findings of the 80 healthy volunteers enrolled in this study are summarized in table 1. Mean age of group one and group two was 31.6 ± 4.5 and 47.2 ± 4.3 years respectively. It was successfully insonated 1020 (98%) of 1040 arteries. Detected blood flow parameters are shown in table 2.

**Table 1:** Demographic and laboratory findings of 80 healthy voluntaries

Factors	Mean ± SD
Age (year)	39.44 ± 8.98
Body mass index (kg/m2)	25 ± 4
Systolic blood pressure (mmhHg)	112.12 ± 13.04
Diastolic blood pressure(mmhHg)	75.16 ± 9.77
Fasting blood sugar (mg/dl)	86.92 ± 8.64
Total Cholesterol (mg/dl)	179.22 ± 2949
HDL Cholesterol (mg/dl)	46.97 ± 12.88
LDL Cholesterol (mg/dl)	105.35 ± 24.88
Triglyceride (mg/dl)	104.00 ± 46.01
Hemglobulin (mg/dl)	14.41 ± 1.37
Hematocrit	43.72 ± 3.66
Blood urea nitrogen (mg/dl)	17.48 ± 8.05
Creatinine (mg/dl)	0.85 ± 0.16

**Table 2:** Flow velocities of main intracranial arteries of both right and left sided

Vessel		**EDV**_**cm/sec**_	**PSV**_**m/sec**_	**MV**_**cm/sec**_	PI	Depth
**MCA**	R	44 ± 8	86 ± 12	58 ± 8	0.81 ± 0.15	52 ± 3
	L	48 ± 10	90 ± 16	62 ± 10	0.76 ± 0.12	53 ± 3
**ACA**	R	40 ± 10	78 ± 14	53 ± 10	0.83 ± 0.15	66 ± 5
	L	41 ± 10	76 ± 16	52 ± 10	0.83 ± 0.17	66 ± 2
**PCA1**	R	32 ± 6	61 ± 11	42 ± 7	0.82 ± 0.18	65 ± 1
	L	34 ± 6	62 ± 10	43 ± 7	0.76 ± 0.16	65 ± 2
**PCA2**	R	34 ± 7	64 ± 10	44 ± 8	0.82 ± 0.16	65 ± 2
	L	34 ± 7	63 ± 10	44 ± 8	0.79 ± 0.13	66 ± 2
**VA3**	R	28 ± 8	49 ± 10	35 ± 8	0.77 ± 0.19	50 ± 7
	L	29 ± 8	51 ± 12	36 ± 9	0.73 ± 0.13	50 ± 7
**VA4**	R	32 ± 8	58 ± 13	41 ± 9	0.77 ± 0.17	66 ± 3
	L	33 ± 8	9 ± 13	42 ± 10	0.74 ± 0.15	66 ± 2
**BAS**		4 ± 8	68 ± 11	48 ± 8	0.82 ± 0.20	95 ± 7

R: Right; L: Left; ACA: Anterior Cerebral artery; MCA: Middle Cerebral Artery; PCA: Posterior Cerebral Artery; VA: Vertebral Artery; BAS: Basilar Artery

EDV: End Diastolic Velocity; PSV: Peak Systolic Velocity; MV: Mean Velocity;

PI: Pulsatility Index

In the anterior cerebral circulation the mean blood flow velocity of MCA was highest, and in the posterior cerebral circulation mean blood flow velocity of basilar artery was higher than vertebral arteries and PCA.

**Figure 1:**
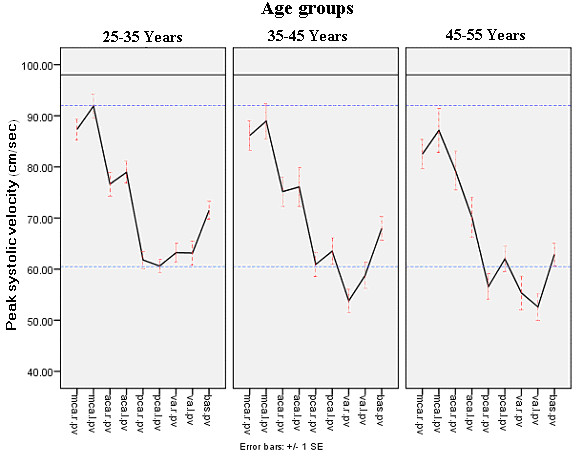
**Peak systolic flow velocity in different cerebral vessels compared for three age groups**. MCA: Middle cerebral artery, PCA: Posterior cerebral artery, ACA: Anterior cerebral artery, VA: Vertebral artey, BA: Basilar artey, PV: Peak systolic velocity, R; Right, L: Left

**Figure 2:**
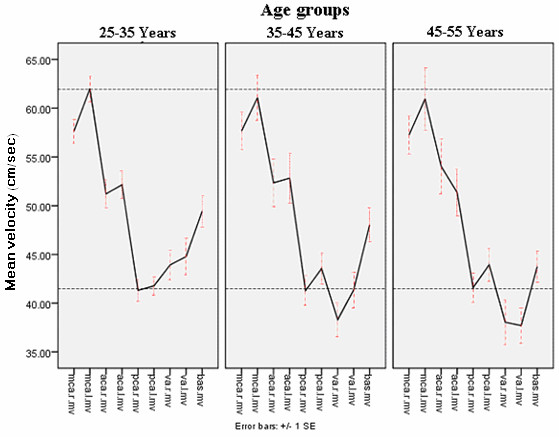
**Mean blood flow velocity in different cerebral vessels compared for three age groups**. MCA: Middle cerebral artery, PCA: Posterior cerebral artery, ACA: Anterior cerebral artery VA: Vertebral artey, BA: Basilar artey, MV: Mean velocity, R; Right, L: Left

There were significant side to side differences of parameters only in both MCAs. Peak systolic blood flow velocity in different cerebral vessels are compared for three age groups in figure 1 and mean velocities of the same vessels are compared in figure 2 showing slight decrease over age in some vessels.

Mean age of men was 40 ± 10 years old and for women was 39 ± 8 years old. The flow velocities tended to be higher in women, however, this difference reached the level of statistical significance only in MCAs (left more than right side).

## Discussion

In our study similar to reported by Rozenkranz, MCA had the highest mean flow velocity and vertebral artery had the lowest mean flow velocity (36 ± 8) [5]. In other studies the anterior circulation vessels usually have higher flow velocities than those of the posterior circulation (MCA > ACA > PCA > BA > VA)[1].

This study showed significant side to side differences in MCAs, being in line with Rosenkranz et al results [5]. Higher values of MV in the left hemispheric arteries were found in Sanches study [6]. That may be due to dominancy of left hemisphere. Macchi et al study found the left side velocity to be higher [7].

Relation of age and blood flow parameters varies from one study to another. For example, Hennerici et al found only a slight decrease in CBFV in the middle cerebral artery but not in the anterior and posterior cerebral arteries [8]. Macchi et al and Bartels et al did not found any age dependency of flow parameters [7,9]. However, most authors in their transcranial Doppler sonographic studies, have found reduction of flow velocities and increase in impedance indices with age [2,5,10-20]. Only slight velocity reduction in middle age and marked reduction beyond the age of 60 years was found. Arnolds et al found a reduction in flow velocities (on average by 20%) in all vessels from the youngest to the oldest group, with a marked reduction after the age of 40 years [11]. Grolimund and Seiler reported a linear decrease in all flow velocities in all examined vessels with increasing age among those having some risk factors [13,14,21,22]. Previous reports demonstrated that age was associated with decreasing flow velocities and increasing pulsatility indices and average decline of 0.3-0.5 per year in mean flow velocities in the individuals between 20-70 years of age[23]. In our study we didn't find relation of age that may be due to lower age variability in our sample.

In our study women tended to show higher velocity values, a finding that is in accordance with other reports although the differences between men and women didn't reach statistical significance [1,8,18,19]. However this result has not been found consistently [24]. The difference has been attributed to generally lower hematocrit in women [8].

Our report is in line with a previous report showing that in premenopausal period, cerebral blood flow is higher among women than in men of the same age [25]. However, after menopause, CBFV in women declines and equals that seen in age-matched men [26]. This may be related, in part, to estrogen levels [27]. Estrogen levels appear to be correlated directly with middle cerebral artery velocity and resistance [28]. Blood velocity and vascular resistance of cerebral microcirculation appear to change according to the phases of a women reproductive life. This may be related, in part to estrogen levels, because of estradiol induced vasodilatation of small cerebral vessels in hypoestrogenic postmenopausal women [27]. The results of different studies are given in the table 3. As shown in the table, cerebral blood flow velocity of our region was relatively higher than other studies. As all the participants were studied using the same device and by one neurologist who is assumed to be the most expert TCD specialist in the area, less measurement variability error is expected. Blood flow velocity difference observed in this study may be explained as: 1-Environmental, social and nutritional factors in this setting may affect the vessel reactivity increasing blood flow velocity. 2- Normal daily variations of the blood velocity may be responsible for part of it. 3- Random error due to smaller sample size may also be considered as a possible explanation. However the authors are more convinced to give higher priority to the first explanation. The findings of this study may be used in better interpretation of TCD results.

**Table 3:** Mean flow velocities of main intracranial arteries in other studies

Study	N	Age range(Y)	Mean age(Y)	**MCA**_**cm/sec**_	**ACA**_**cm/sec**_	**PCA**_**cm/sec**_
**US**^**29**^	50	20-65	36	62 ± 12	51 ± 12	44 ± 11
**Italy**^**30**^	40	25-60	37	65 ± 13	48 ± 20	35 ± 18
**Germany**^**31**^	50	< 40	-	58 ± 8	47 ± 14	41 ± 9
		40-60	-	58 ± 12	53 ± 1	-
**Netherland**^**32**^	120	30-40	-	67 ± 8	-	-
		40-50	-	68 ± 11	-	-
**Norway**^**33**^	20	20-35	28	67 ± 7	-	-
**Poland**^**34**^	182	20-40	-	81 ± 20	56 ± 14	52 ± 12
		41-60	-	73 ± 19	53 ± 16	51 ± 12
		> 60	-	59 ± 11	40 ± 9	40 ± 9
**North-West of Iran**	80	25-40	32 ± 4	89 ± 12	79 ± 10	69 ± 8
		40-60	47 ± 4	87 ± 14	77 ± 14	62 ± 10

## Competing interests

The authors declare that they have no competing interests.

## Authors' contributions

**MF**: designing of study & performing of Doppler studies & as corresponding author. **SNK**: collecting of the volunteers & their data & writing the first draft of the article. **HSB**: final analysis of data & preparing the figures & English editing & first submission
